# Inflammatory rheumatic diseases and the risk of Parkinson's disease: A systematic review and meta-analysis

**DOI:** 10.3389/fneur.2022.999820

**Published:** 2022-11-10

**Authors:** Lili He, Hecong Zhao, Fuli Wang, Xiaoyan Guo

**Affiliations:** Department of Neurology, The Affiliated Hospital of Southwest Medical University, Luzhou, China

**Keywords:** Parkinson's disease, inflammatory rheumatic diseases, risk, systematic review, meta-analysis

## Abstract

**Background:**

Several studies showed inconsistencies in the relationships between inflammatory rheumatic diseases (IRDs) and the risk of Parkinson's disease (PD). Therefore, we carried out a meta-analysis to investigate the associations between different IRDs and PD risk.

**Methods:**

A comprehensive search was undertaken on PubMed, Embase, Cochrane Library, and Web of Science databases up to June 2022. Studies reporting the relationships between IRDs and PD risk were included. Pooled relative risks (RRs) with 95% confidence intervals (CIs) were calculated by using random-effects models.

**Results:**

Twenty-two publications covering seven IRDs containing data from 833,004 patients were identified for quantitative analysis. The pooled results indicated that ankylosing spondylitis (RR = 1.55, 95% CI: 1.31–1.83, I^2^ = 32.1%, *P* < 0.001), Sjögren's syndrome (RR = 1.34, 95% CI: 1.22–1.47, I^2^ = 58.5%, *P* < 0.001), and Behcet's disease (RR = 1.93, 95% CI: 1.07–3.49, I^2^ = 57.6%, *P* = 0.030) were associated with an increased PD risk. However, no significant associations were observed between gout, rheumatoid arthritis, systemic lupus erythematosus, as well as polymyalgia rheumatica and the subsequent development of PD.

**Conclusion:**

Ankylosing spondylitis, Sjögren's syndrome, and Behcet's disease may increase PD risk.

## Introduction

Parkinson's disease (PD) is a progressive neurodegenerative disorder that causes substantial motor impairments such as resting tremor, bradykinesia, rigidity, and postural instability, as well as a series of non-motor symptoms (1, 2). The main pathological changes of PD are the progressive loss of dopaminergic neurons in the substantia nigra, along with the deposition of synuclein, also known as Lewy bodies (3). Aging, environmental, genetic, and lifestyle factors seem to be involved in the formation of underlying etiologies (4, 5). However, the exact mechanisms leading to programmed dopamine death in PD are still unknown (6). It has been suggested that chronic inflammation may play a crucial role in the pathogenesis of PD (7).

Inflammatory rheumatic diseases (IRDs) encompass a wide range of conditions, including chronic inflammatory arthritis such as rheumatoid arthritis (RA), gout, and ankylosing spondylitis (AS). It also contains vasculitis and connective tissue disorders, like Sjögren's syndrome (SS) and systemic lupus erythematosus (SLE). This highly heterogeneous group of disorders was characterized by persistent systemic inflammation mainly affecting the musculoskeletal system and connective tissue (8–10). Previous studies have demonstrated that IRDs were associated with an increased risk of dementia, depressive disorders, and stroke (11–13). Furthermore, several articles have attempted to explore the correlation between IRDs and PD risk. However, these findings are inconsistent (14–18). Therefore, a meta-analysis is warranted to synthesize these results and further elucidate the association between IRDs and PD risk.

## Methods

### Search strategy

This analysis study was conducted based on the Preferred Reporting Items for Systematic Reviews and Meta-analysis guidelines (PRISMA) (19). Two researchers (LLH and FLW) independently searched relevant articles published in the PubMed, Embase, Cochrane Library, and Web of Science databases up to June 2022. The following search terms were used with restriction to English: “inflammatory rheumatic disease”, “rheumatoid arthritis”, “systemic lupus erythematosus”, “ankylosing spondylitis”, “Sjögren's syndrome”, “systemic sclerosis”, “myositis”, “dermatomyositis”, “polymyositis”, “axial spondyloarthritis”, “psoriatic arthritis”, “arthritis, reactive”, “systemic vasculitis”, “giant cell arteritis”, “temporal arteritis”, “Takayasu's arteritis”, “granulomatosis with polyangiitis”, “Churg Strauss syndrome”, “Behcet Syndrome”, “gout”, and “Parkinson disease”. In addition, we manually screened the references of articles to identify additional eligible studies.

### Inclusion and exclusion criteria

The inclusion criteria were as follows: (1) Studies reporting relationships between PD risk and IRDs as abovementioned; (2) case–control, cross-sectional, or cohort designs; (3) studies presenting a measure of association (such as an odds ratio [OR], relative risk [RR], hazard ratio [HR]), standardized incidence ratio [SIR], or incidence rate ratio [IRR]) for the association between IRDs and PD risk, with 95% confidence interval (CI).

Exclusion criteria included (1) case reports, letters, reviews, conference abstracts, and editorials; (2) animal and *in vitro* studies.

### Quality assessment and data extraction

For the cohort and case–control studies, we adopted the Newcastle–Ottawa Quality Assessment Scale (NOS) to assess the quality of the studies (20). For cross-sectional studies, we used the Agency for Healthcare Research and Quality (AHRQ) to detect the bias in the studies (21). Two investigators (LLH and HCZ) independently evaluated the included studies and extracted relevant information such as first author, publication year, different types of IRDs, study populations, study designs, duration of the study, diagnosis criteria of PD and IRDs, effect estimates with 95% CIs, and adjusted variables (e.g., age, comorbidities, sex, region, medication, chronic obstructive, tobacco consumption, socioeconomic status, and body mass index). Any discrepancies were resolved by reaching a consensus or rechecking the original literature data.

### Statistical analysis

Stata 15.0 software was used to analyze the data. Adjusted effect estimates with corresponding 95% CIs for the association between different IRDs and PD risk were chosen as the primary endpoints of the interest of pooling. The risk estimate measures involved (OR, RR, HR, IRR, and SIR) were considered equivalent (22). Heterogeneity was estimated by using I^2^ statistic. We used the fixed-effects model for pooled analysis when I^2^ < 50% and *P* ≥ 0.1, whereas the random-effects model was chosen when I^2^ ≥ 50% or *P* < 0.1 due to the relatively significant heterogeneity. Subgroup analyses were performed to investigate the potential heterogeneity. Sensitivity analyses were conducted to check the stability of outcomes by eliminating each study in turn. Finally, publication bias was conducted through Begg's test. A *P*-value < 0.05 showed the existence of publication bias (23).

## Results

### Eligible studies

**Figure 1:**
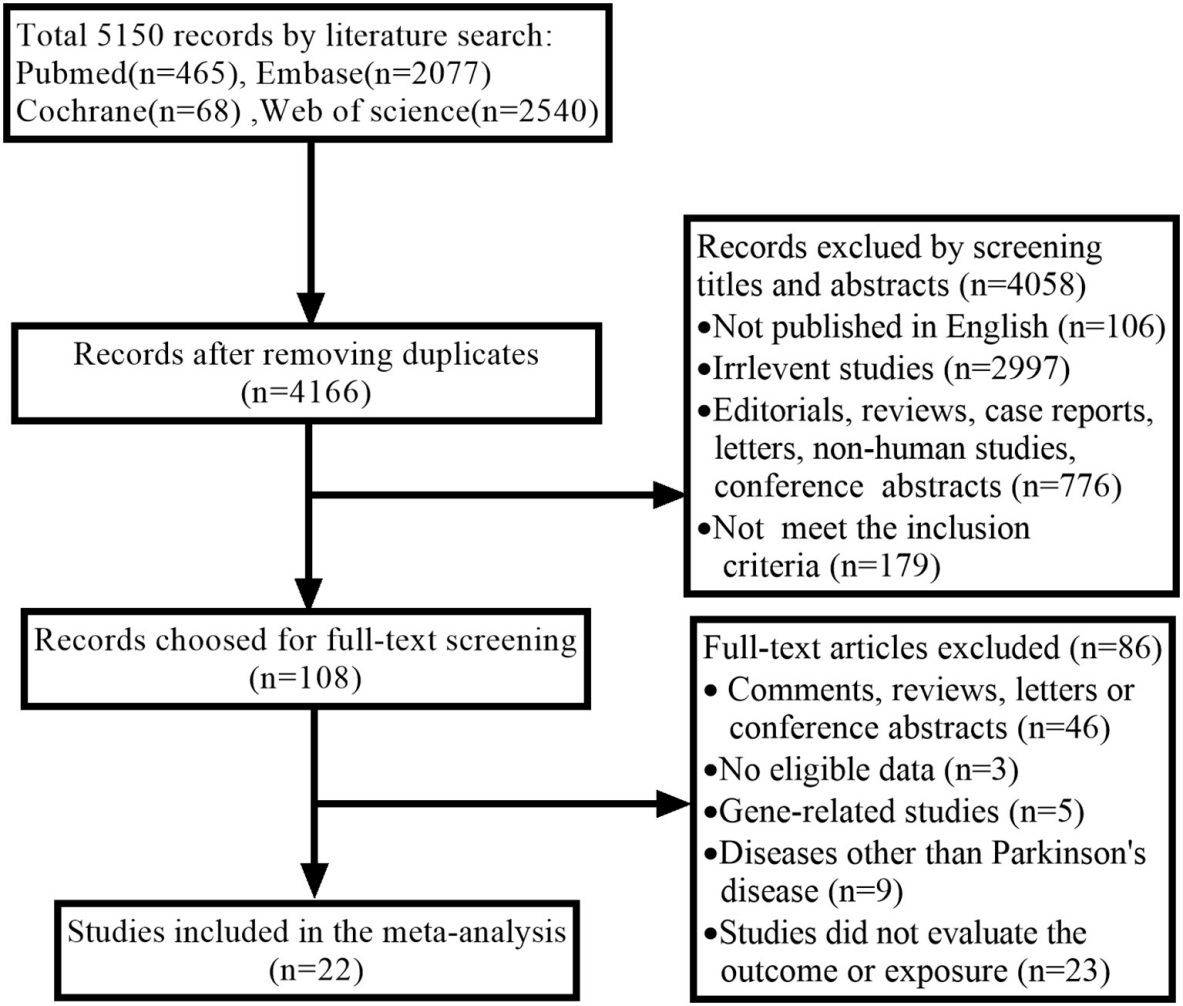
Flowchart of the study selection.

A comprehensive search yielded a total of 5,150 articles. We removed 5,042 articles due to duplicated documents, unmatched titles, and unmatched abstracts. Following a review of the remaining 108 full-text articles, twenty-two articles were found to meet the inclusion criteria and were included in the current meta-analysis (14–18, 24–40). The process of selection is described in Figure 1.

### Study characteristics

The qualitative analysis included twenty-two observational studies (fourteen cohort designs, one cross-sectional design, and seven case–control designs) reporting associations between seven types of IRDs and the subsequent development of PD. In total, 833,004 patients from three continents (Europe, Asia, and North America) and eight countries are involved in the study. The characteristics of the eligible studies are summarized in Table 1.

**Table 1:** Characteristics of included studies.

**Studies**	**Diseases**	**Countries**	**Study design**	**Study period**	**Patients**	**Controls**	**Effect size**	**Adjusted risk**
Alonso et al. (26)	Gout	England	Case-control	1995–2001	1,052	6,634	OR	0.69 (0.48–0.99)
De Vera et al. (27)	Gout	Canada	Cohort	1991–2004	11,258	56,199	RR	0.70 (0.59–0.83)
Rugbjerg et al. (25)	RA/ PMR	Denmark	Case-control	1986–2006	13,695	68,445	OR	0.70 (0.50–0.90)/ 1.00 (0.80–1.30)
Li et al. (15)	RA	Sweden	Cohort	1964–2007	52,994	NA	SIR	1.07 (0.89–1.26)
Li et al. (15)	AS	Sweden	Cohort	1964–2007	5,402	NA	SIR	1.51 (0.82–2.53)
Li et al. (15)	SS	Sweden	Cohort	1964–2007	1,360	NA	SIR	2.01 (0.63–4.72)
Li et al. (15)	SLE	Sweden	Cohort	1964–2007	5,677	NA	SIR	1.00 (0.43–1.97)
Li et al. (15)	BD	Sweden	Cohort	1964–2007	2,718	NA	SIR	1.33 (0.63–2.45)
Li et al. (15)	PMR	Sweden	Cohort	1964–2007	20,110	NA	SIR	1.25 (1.01–1.53)
Schernhammer et al. (17)	Gout	Denmark	Case-control	2001–2008	4,484	22,416	OR	1.06 (0.90–1.25)
Lai et al. (28)	Gout	China	Case-control	2000–2010	3,854	15,416	OR	1.00 (0.90–1.11)
Liu et al. (29)	SLE	China	Cohort	2000–2010	12,817	51,268	HR	0.68 (0.51–0.90)
Pakpoor et al. (30)	Gout	England	Cohort	1999–2012	214,653	9,000,000	RR	1.11 (1.05–1.17)
Sung et al. (14)	RA	China	Cohort	1998–2010	33,221	132,884	HR	0.65 (0.58–0.73)
Wu et al. (31)	SS/AS	China	Case-control	2000–2010	7,716	75,129	OR	1.38 (1.15–1.66)/ 1.20 (0.92–1.57)
Chang et al. (24)	RA	China	Cohort	2001–2012	1,954	NA	HR	1.14 (1.03–1.28)
Chang et al. (24)	SLE	China	Cohort	2001–2012	3,055	NA	HR	1.21 (0.91–1.61)
Chang et al. (24)	SS	China	Cohort	2001–2012	8,422	NA	HR	1.56 (1.35–1.79)
Ju et al. (32)	SS	China	Cohort	2000–2010	12,640	50,560	HR	1.23 (1.16–1.30)
Park et al. (40)	BD	Korean	Cohort	2010–2013	11,525	34,575	HR	2.47 (1.65–3.68)
Singh et al. (33)	Gout	England	Cohort	2006–2012	1,129	21,507	HR	1.13 (1.07–1.21)
Hsu et al. (34)	SS	China	Cohort	2000–2014	17,028	68,094	HR/IRR	1.23 (1.07–1.42)/1.37 (1.19–1.57)
Hu et al. (18)	Gout	China	Cohort	2000–2000	7,900	7,900	RR/HR	1.36 (1.15–1.60)/1.01 (0.93–1.31)
Yeh et al. (35)	AS	China	Cohort	2000–2010	6,440	25,760	HR	1.75 (1.38–2.22)
Bacelis et al. (16)	RA	Sweden	Case-control	1964–2017	4,819	48,190	OR	0.47 (0.28–0.75)
Kim et al. (36)	Gout	Korean	Cohort	2002–2019	327,160	327,160	IRR/HR	0.98 (0.89–1.07)/1.00 (0.91–1.10)
Yoon et al. (39)	AS	Korea	Cohort	2009–2019	18,210	72,840	HR	1.82 (1.38–2.39)
Pou et al. (37)	Gout	Spanish	Case-control	2010–2019	17,629	70,516	OR	0.83 (0.76–0.91)
Watad et al. (38)	AS	Israel	Cross-sectional	2002–2016	4,082	20,397	OR	1.49 (1.05–2.13)

RA, rheumatoid arthritis; SLE, systemic lupus erythematous; SS, Sjogren's syndrome; AS, ankylosing spondylitis; PMR, polymyalgia rheumatica; BD, Behcet's disease; HR, hazard ratio; IRR, incidence rate ratio; OR, odds ratio; RR, relative risk; SIR, standardized incidence ratio; NA, not application.

### Quality assessment of all included studies

Among twenty-two studies, one study showed higher quality (nine stars at the NOS), twenty were moderate quality (nineteen studies ranked at seven–eight stars at the NOS, one study ranked at eight scores by the AHRQ checklist), and one study was low quality (less than seven stars at the NOS). Studies above six scores were considered to have a low risk of bias (Supplementary Tables S1a,b).

### Overall meta-analysis

**Figure 2:**
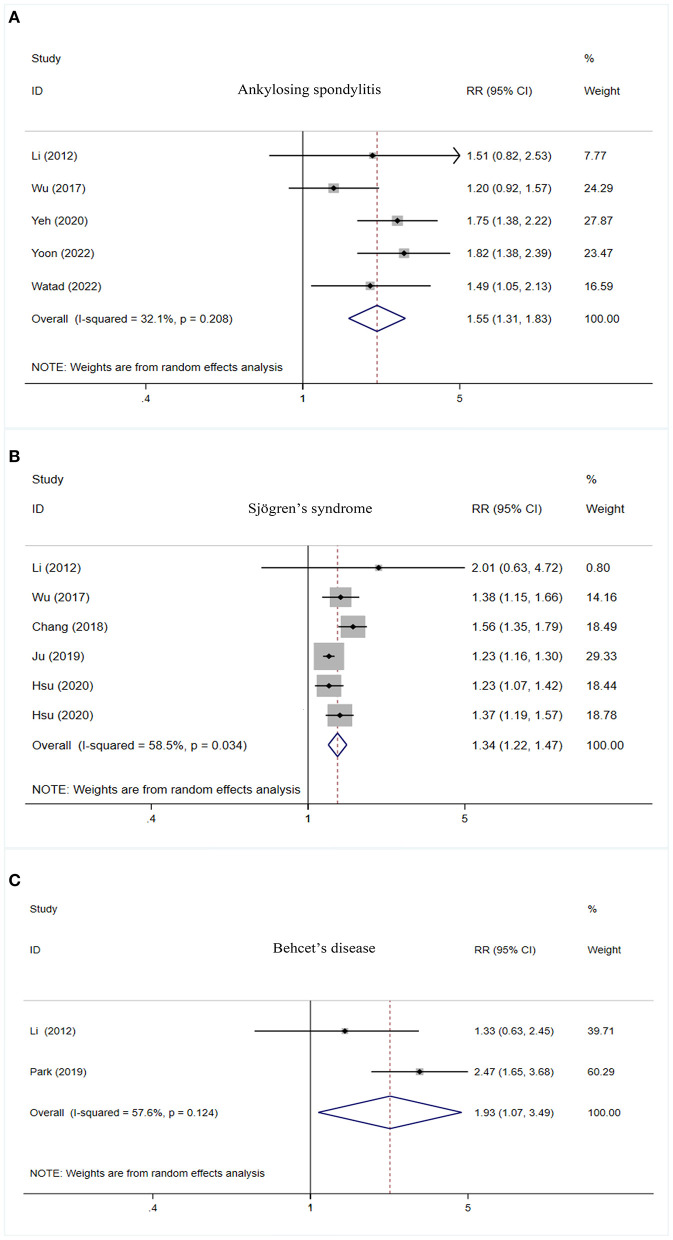
Forest plot of PD risk in patients with ankylosing spondylitis **(A)**, Sjögren's syndrome **(B)**, and Behcet's disease **(C)**. PD, Parkinson's disease; CI, confidence interval; RR, relative risk.

Higher risk of PD was observed in cases with ankylosing spondylitis (RR = 1.55, 95% CI: 1.31–1.83, I^2^ = 32.1%, *P* < 0.001), Sjögren's syndrome (RR = 1.34, 95% CI: 1.22–1.47, I^2^ = 58.5%, *P* < 0.001), and Behcet's disease (RR = 1.93, 95% CI: 1.07–3.49, I^2^ = 57.6%, *P* = 0.030) (Table 2, Figure 2).

**Table 2:** Overall meta-analysis results on associations between different types of inflammatory rheumatic diseases and PD risk.

**Diseases**	**Number of studies**	**RR (95% CI)**	***P*-value**	**I^2^ (%)**	**P for heterogeneity**	**Begg's test**
Ankylosing spondylitis	5	1.55 (1.31–1.83)	< 0.001^*^	32.1	0.208	1.000
Sjögren's syndrome	5	1.34 (1.22–1.47)	< 0.001^*^	58.5	0.034	0.707
Behcet's disease	2	1.93 (1.07–3.49)	0.030^*^	57.6	0.124	1.000
Gout	9	0.99 (0.91–1.08)	0.847	86.7	< 0.001	0.876
Rheumatoid arthritis	5	0.79 (0.58–1.08)	0.142	93.5	< 0.001	0.462
Systemic lupus erythematosus	3	0.91 (0.60–1.37)	0.647	74.6	0.020	1.000
Polymyalgia rheumatica	2	1.13 (0.91–1.40)	0.277	46.6	0.171	1.000

PD, Parkinson's disease; CI, confidence interval; RR, relative risk;

* statistically significant differences.

**Figure 3:**
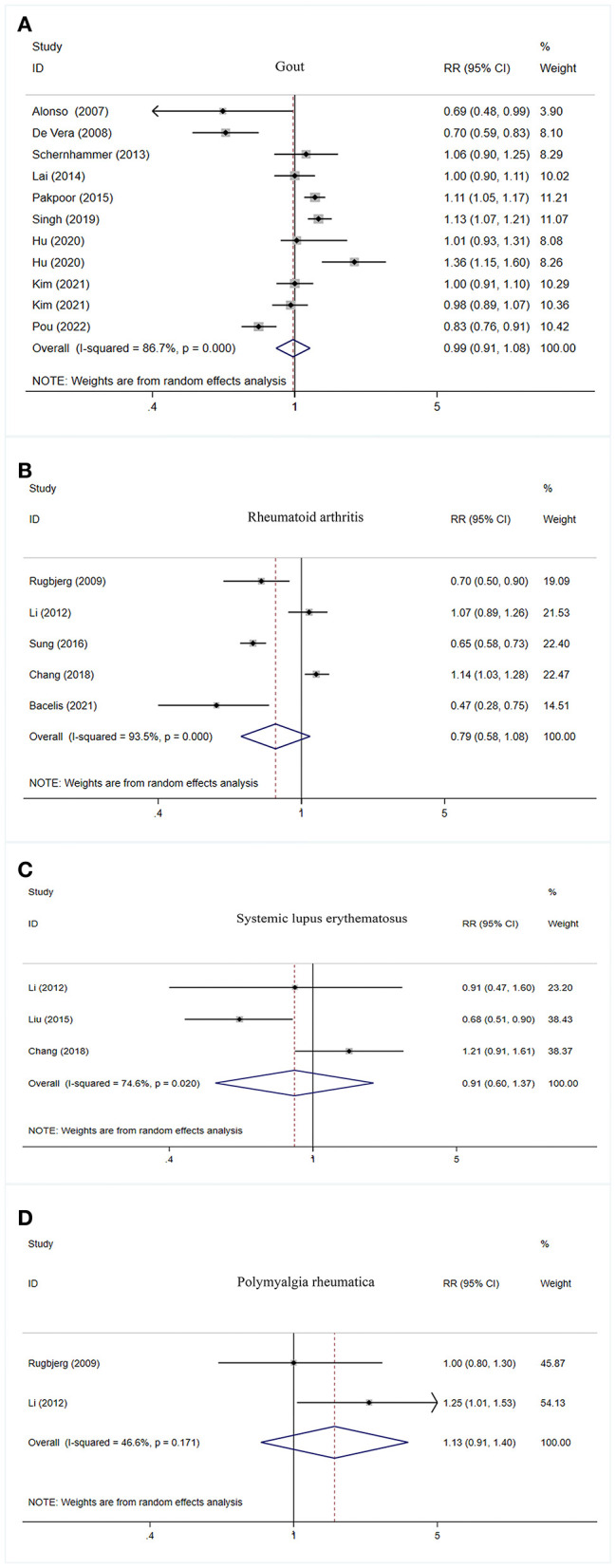
Forest plot of PD risk in patients with gout **(A)**, rheumatoid arthritis **(B)**, systemic lupus erythematosus **(C)**, and polymyalgia rheumatic **(D)**. PD, Parkinson's disease; CI, confidence interval; RR, relative risk.

No significant association was observed between gout, rheumatoid arthritis, systemic lupus erythematosus, as well as polymyalgia rheumatica and PD risk (Table 2, Figure 3).

Significant heterogeneities were found, so we pooled results of ankylosing spondylitis, Sjögren's syndrome, rheumatoid arthritis, and gout by using random-effects models and proceed to explore the potential discrepancies in terms of genders, study designs, effect sizes, and regions. We did not conduct subgroup analysis for systemic lupus erythematosus, Behcet's disease, and polymyalgia rheumatica due to the limited number.

### Subgroup analysis based on gender

Ankylosing spondylitis increased PD risk in both male patients (RR = 1.76, 95% CI: 1.39–2.22, *P* < 0.001) and female patients (RR = 1.80, 95% CI: 1.35–2.39, *P* < 0.001). Female patients (RR = 1.28, 95% CI: 1.21–1.35, *P* < 0.001) with Sjögren's syndrome had a higher risk of PD. There was a trend of decreased risk of PD in patients with rheumatoid arthritis in both male patients (RR = 0.61, 95% CI: 0.49–0.76, *P* < 0.001) and female patients (RR = 0.58, 95% CI: 0.38–0.89, *P* = 0.013). The detailed results of the gender subgroup analyses are listed in Supplementary Table S2 and Supplementary Figures S1–S4.

### Subgroup analysis based on the study design

Ankylosing spondylitis patients had a higher risk of PD, as shown in cohort studies (RR = 1.75, 95% CI: 1.48–2.08, *P* < 0.001) and cross-sectional designs (RR = 1.49, 95% CI: 1.05–2.12, *P* = 0.027). Sjögren's syndrome showed an increased risk of PD in both cohort studies (RR = 1.33, 95% CI: 1.20–1.48, *P* < 0.001) and case–control studies (RR = 1.38, 95% CI: 1.15–1.66, *P* = 0.001). Rheumatoid arthritis was associated with a decreased risk of PD in case–control designs (RR = 0.6, 95% CI: 0.41–0.88, *P* = 0.009). The results of the subgroup analyses based on the study design are presented in Supplementary Table S3 and Supplementary Figures S5–S8.

### Subgroup analysis based on effect size

Ankylosing spondylitis had an increased PD risk by using both “HR” (RR = 1.78, 95% CI: 1.49–2.13, *P* < 0.001) and “OR” (RR = 1.30, 95% CI: 1.05–1.61, *P* = 0.016) as effect sizes. Patients with Sjögren's syndrome had a higher PD incidence when the effect size was estimated by “OR” (RR = 1.38, 95% CI: 1.15–1.66, *P* = 0.010), “HR” (RR = 1.32, 95% CI: 1.15–1.52, *P* < 0.001), and “IRR” (RR = 1.37, 95% CI: 1.19–1.57, *P* < 0.001). Rheumatoid arthritis was associated with a decreased risk of PD when using “OR” as the assessment criterion (RR = 0.60, 95% CI: 0.41–0.88, *P* = 0.009). The results of the subgroup analyses based on effect size are listed in Supplementary Table S4 and Supplementary Figures S9–S12.

### Subgroup analysis based on region

Patients in Asia with ankylosing spondylitis (RR = 1.55, 95% CI: 1.28–1.89, *P* = 0.001) and Sjögren's syndrome (RR = 1.33, 95% CI: 1.22–1.46, *P* < 0.001) had a higher risk of PD. Patients with gout in North America had a lower risk of PD (RR = 0.70, 95% CI: 0.59–0.83, *P* < 0.001). Detailed results are shown in Supplementary Table S5 and Supplementary Figures S13–S16.

### Sensitivity analysis and publication bias

Sensitivity analyses were carried out by eliminating one study in turn to evaluate the stability and reliability of the individual outcome on the overall analysis. Sensitivity analyses demonstrated that the pooled RRs with 95% CIs were not affected by any individual study. It confirmed the consistency and dependability of our findings (Supplementary Figures S17–S23). Potential publication bias was assessed by Begg's test (P_AS_ = 1.000; P_SS_ = 0.707; P_BD_ = 1.000; P_Gout_ = 0.876; P_RA_ = 0.462; P_SLE_ = 1.000; P_PMR_ = 1.000), and no significant publication bias was detected (Supplementary Figures S24–S30).

## Discussion

This meta-analysis is the first comprehensive review to investigate the associations between IRDs and PD risk. The results suggest that ankylosing spondylitis, Sjögren's syndrome, and Behcet's disease may increase the risk of PD.

The exact mechanisms are unclear. There are some possible explanations. First, systemic inflammation involving in neuroinflammation may contribute to the pathogenesis of PD through cytokine-induced inflammatory responses or abnormal immune responses (41). Accumulating evidence showed that the major products of IRDs-peripheral cytokine may cross the blood–brain barrier directly through the leaky areas of blood–brain barrier (such as damaged tight junctions or circumventricular organs) or through the pathway of receptor-mediated transcytosis (42). Peripheral cytokine reaching the brain can activate the microglia and upregulate inflammatory response, thus leading to loss of dopaminergic neurons (42–44). Peripheral cytokine can also have an indirect impact on brain signals by stimulating peripheral afferent nerves, which can trigger strong responses of neurodegenerative processes (45). Furthermore, peripheral cytokine may activate the inflammasomes, such as nucleotide-binding oligomerization domain-like receptor protein 3, which can promote the maturation of interleukin IL-1β and IL-18, thus accelerating neurodegeneration (46). Meanwhile, reactive oxygen species and oxidative stress generated by inflammation may also contribute to the damage of dopamine neurons in PD (43).

Second, several types of research have manifested a connection between physical inactivity and the subsequent development of PD (47). Patients with IRDs may have less physical activities due to the symptoms of IRDs, such as arthritis, pain, and fatigue, which may play a role in the susceptibility to PD.

In agreement with Ungprasert's findings (48), we found that gout showed no correlation with PD risk. We speculate that the gout-related inflammation may be offset by the neuroprotective effect of hyperuricemia due to its antioxidant property in gout (48). However, no significant association between rheumatoid arthritis and PD risk was observed, which was not consistent with Li's results (49). We included more comprehensive studies and sample sizes in the current analysis may explain the inconsistent findings.

Gender subgroup analysis indicated rheumatoid arthritis might decrease PD risk. Previous studies showed that high levels of lysosomal cathepsin D released by rheumatoid arthritis may reduce aggregation of α-synuclein (50). Therefore, it may play a neuroprotective role in the development of PD through the lysosome pathway (51–53). Moreover, higher frequent using non-steroidal anti-inflammatory drugs, particularly ibuprofen (54) to relieve arthritis in patients with rheumatoid arthritis, may play a part role in decreasing PD risk.

## Strengths and limitations

The current meta-analysis has the following strengths. First, the majority of included studies have relatively high quality and large sample sizes, which provide more reliable sources of evidence. Second, subgroup analyses based on the stratification factors are carried out.

Nevertheless, there are some limitations. First, most of the studies relied on diagnosis codes from medical record databases. Inconsistencies of diagnostic criteria of IRDs and PD may create ascertainment bias. Second, language bias should be considered because only studies published in English were included. Third, for Behcet's disease, there were only two studies included in the current meta-analysis; therefore, the statistical power for the results of Behcet's disease may be not sufficient enough. More studies are needed to verify the conclusion that Behcet's disease may increase PD risk. Fourth, unconsidered or unmeasured variables influencing the findings of the included original studies may give rise to some bias. Thus, our results may be explained with cautions.

## Conclusion

In summary, this systematic review and meta-analysis indicate that patients with ankylosing spondylitis, Sjögren's syndrome, and Behcet's disease may have a higher risk of PD. More prospective studies are needed to verify our findings.

## Data availability statement

The original contributions presented in the study are included in the article/Supplementary material, further inquiries can be directed to the corresponding author/s.

## Author contributions

LH and XG participated in design. LH and FW have independently screened the literature. LH and HZ were involved in collecting and analyzing data. This article was written by LH, with revisions by XG. All authors read and approved the final manuscript.

## Conflict of interest

The authors declare that the research was conducted in the absence of any commercial or financial relationships that could be construed as a potential conflict of interest.

## Publisher's note

All claims expressed in this article are solely those of the authors and do not necessarily represent those of their affiliated organizations, or those of the publisher, the editors and the reviewers. Any product that may be evaluated in this article, or claim that may be made by its manufacturer, is not guaranteed or endorsed by the publisher.
