# Supplementary material for: Inflammatory rheumatic diseases and the risk of Parkinson's disease: A systematic review and meta-analysis
Source: Front Neurol. 2022 Nov 10;13:999820. doi: 10.3389/fneur.2022.999820 (PMC9684169; doi:10.3389/fneur.2022.999820)

**Supplementary**

# Table legend

**Supplementary Table S1** Risk of bias assessment using the Newcastle-Ottawa Quality Assessment Scale (NOS) (**Table S1a**) and the Agency for Healthcare Research and Quality (AHRQ) chicks (**Table S1b**).

**Supplementary Table S2** The results of the subgroup analysis based on gender.

**Supplementary Table S3** The results of the subgroup analysis based on the study design.

**Supplementary Table S4** The results of the subgroup analysis based on effect size.

**Supplementary Table S5** The results of the subgroup analysis based on region.

# Figure legend

# Subgroup analysis

**Supplementary Figure S1** Forest plot of gender-subgroup analysis for association between ankylosing spondylitis and PD risk.

**Supplementary Figure S2** Forest plot of gender-subgroup analysis for association between Sjögren’s syndrome and PD risk.

**Supplementary Figure S3** Forest plot of gender-subgroup analysis for association between rheumatoid arthritis and PD risk.

**Supplementary Figure S4** Forest plot of gender-subgroup analysis for association between gout and PD risk.

**Supplementary Figure S5** Forest plot of study design’s subgroup analysis for association between ankylosing spondylitis and PD risk.

**Supplementary Figure S6** Forest plot of study design’s subgroup analysis for association between Sjögren’s syndrome and PD risk.

**Supplementary Figure S7** Forest plot of study design’s subgroup analysis for association between rheumatoid arthritis and PD risk.

**Supplementary Figure S8** Forest plot of study design’s subgroup analysis for association between gout and PD risk.

**Supplementary Figure S9** Forest plot of effect size’s subgroup analysis for association between ankylosing spondylitis and PD risk.

**Supplementary Figure S10** Forest plot of effect size’s subgroup analysis for association between Sjögren’s syndrome and PD risk.

**Supplementary Figure S11** Forest plot of effect size’s subgroup analysis for association between rheumatoid arthritis and PD risk.

**Supplementary Figure S12** Forest plot of effect size’s subgroup analysis for association between gout and PD risk.

**Supplementary Figure S13** Forest plot of region-subgroup analysis for association between ankylosing spondylitis and PD risk.

**Supplementary Figure S14** Forest plot of region-subgroup analysis for association between Sjögren’s syndrome and PD risk.

**Supplementary Figure S15** Forest plot of region-subgroup analysis for association between rheumatoid arthritis and PD risk.

**Supplementary Figure S16** Forest plot of region-subgroup analysis for association between gout and PD risk.

# Sensitivity analysis

**Supplementary Figure S17** Sensitivity analysis of studies on ankylosing spondylitis for PD risk.

**Supplementary Figure S18** Sensitivity analysis of studies on Sjögren’s syndrome for PD risk.

**Supplementary Figure S19** Sensitivity analysis of studies on Behcet’s disease for PD risk.

**Supplementary Figure S20** Sensitivity analysis of studies on gout for PD risk.

**Supplementary Figure S21** Sensitivity analysis of studies on rheumatoid arthritis for PD risk.

**Supplementary Figure S22** Sensitivity analysis of studies on systemic lupus erythematosus for PD risk.

**Supplementary Figure S23** Sensitivity analysis of studies on polymyalgia rheumatica for PD risk.

# Publication bias

**Supplementary Figure S24** Publication bias analysis of studies on ankylosing spondylitis for PD risk estimated by Begger’s test.

**Supplementary Figure S25** Publication bias analysis of studies on Sjögren’s syndrome for PD risk estimated by Begger’s test.

**Supplementary Figure S26** Publication bias analysis of studies on Behcet’s disease for PD risk estimated by Begger’s test.

**Supplementary Figure S27** Publication bias analysis of studies on gout for PD risk estimated by Begger’s test.

**Supplementary Figure S28** Publication bias analysis of studies on rheumatoid arthritis for PD risk estimated by Begger’s test.

**Supplementary Figure S29** Publication bias analysis of studies on systemic lupus erythematosus for PD risk estimated by Begger’s test.

**Supplementary Figure S30** Publication bias analysis of studies on polymyalgia rheumatica for PD risk estimated by Begger’s test.

**Table S1a** Newcastle-Ottawa Quality Assessment Scale for twenty-one studies included in this meta-analysis.

| **Source** | **Study Design** | **Selection** | **Comparability** | **Exposure/Outcome** | **Scores** |
| --- | --- | --- | --- | --- | --- |
| Alonso et al, 2007 | Case-control | 3 | 2 | 2 | 7 |
| De Vera et al, 2008 | Cohort | 4 | 2 | 3 | 9 |
| Rugbjerg et al, 2009 | Case-control | 3 | 1 | 3 | 7 |
| Li et al, 2012 | Cohort | 3 | 2 | 3 | 8 |
| Schernhammer et al, 2013 | Case-control | 3 | 2 | 2 | 7 |
| Lai et al, 2014 | Case-control | 4 | 1 | 2 | 7 |
| Liu et al, 2015 | Cohort | 4 | 2 | 2 | 8 |
| Pakpoor et al, 2015 | Cohort | 3 | 1 | 2 | 6 |
| Sung et al, 2016 | Cohort | 4 | 2 | 2 | 8 |
| Wu et al, 2017 | Case-control | 4 | 2 | 2 | 8 |
| Chang et al, 2018 | Cohort | 3 | 2 | 2 | 7 |
| Ju et al, 2019 | Cohort | 4 | 2 | 2 | 8 |
| Park et al, 2019 | Cohort | 4 | 2 | 2 | 8 |
| Singh et al, 2019 | Cohort | 3 | 2 | 2 | 7 |
| Hsu et al, 2020 | Cohort | 4 | 2 | 2 | 8 |
| Hu et al, 2020 | Cohort | 4 | 2 | 2 | 8 |
| Yeh et al, 2020 | Cohort | 4 | 2 | 2 | 8 |
| Bacelis et al, 2021 | Case-control | 4 | 1 | 2 | 7 |
| Kim et al, 2021 | Cohort | 4 | 2 | 2 | 8 |
| Yoon et al, 2022 | Cohort | 4 | 2 | 2 | 8 |
| Pou et al, 2022 | Case-control | 4 | 2 | 2 | 8 |

**Table S1b** Agency for Healthcare Research and Quality (AHRQ) checklist for one study included in this meta-analysis.

| **Items** | **Yes** | **No** | **Unclear** |
| --- | --- | --- | --- |
| 1.Define the source of information (survey, record review). | 1 |  |  |
| 2.List inclusion and exclusion criteria for exposed and unexposed subjects (cases and controls) or refer to previous publications. | 1 |  |  |
| 3.Indicate time period used for identifying patients. | 1 |  |  |
| 4.Indicate whether or not subjects were consecutive if not population-based. | 1 |  |  |
| 5.Indicate if evaluators of subjective components of study were masked to other aspects of the status of the participants. | 1 |  |  |
| 6.Describe any assessments undertaken for quality assurance purposes (e.g., test/retest of primary outcome measurements). | 1 |  |  |
| 7.Explain any patient exclusions from analysis. | 1 |  |  |
| 8.Describe how confounding was assessed and/or controlled. | 1 |  |  |
| 9.If applicable, explain how missing data were handled in the analysis. |  | 0 |  |
| 10.Summarize patient response rates and completeness of data collection. |  | 0 |  |
| 11.Clarify what follow-up, if any, was expected and the percentage of patients for which incomplete data or follow-up was obtained. |  |  | 0 |
| Total | 8 |  |  |

**Table S2** The results of the subgroup analysis based on gender.

| **Diseases** | **Subgroups** | **Number of studies** | **RR (95% CI)** | **P-value** | **I^2^ (%)** | **P for heterogeneity** |
| --- | --- | --- | --- | --- | --- | --- |
| Ankylosing spondylitis | Male | 2 | 1.76 (1.39-2.22) | <0.001* | 0.0 | 0.635 |
|  | Female | 2 | 1.80 (1.35-2.39) | <0.001* | 0.0 | 0.785 |
| Sjögren’s syndrome | Male | 2 | 1.00 (0.87-1.16) | 0.962 | 0.0 | 0.920 |
|  | Female | 2 | 1.28 (1.21-1.35) | <0.001* | 0.0 | 1.000 |
| Rheumatoid arthritis | Male | 2 | 0.61 (0.49-0.76) | <0.001***** | 0.0 | 0.933 |
|  | Female | 2 | 0.58 (0.38-0.89) | 0.013* | 48.4 | 0.164 |
| Gout | Male | 9 | 0.98 (0.89-1.08) | 0.688 | 74.3 | <0.001 |
|  | Female | 9 | 1.10 (1.00-1.22) | 0.056 | 44.8 | 0.070 |

CI, confidence interval; RR, relative risk; * Statistically significant differences

**Table S3** The results of the subgroup analysis based on the study design.

| **Diseases** | **Subgroups** | **Number of studies** | **RR (95% CI)** | **P-value** | **I^2^ (%)** | **P for heterogeneity** |
| --- | --- | --- | --- | --- | --- | --- |
| Ankylosing spondylitis | Case-control | 1 | 1.20 (0.92-1.57) | 0.181 | NA | NA |
|  | Cohort | 3 | 1.75 (1.48-2.08) | <0.001* | 0.00 | 0.843 |
|  | Cross-sectional | 1 | 1.49 (1.05-2.12) | 0.027* | NA | NA |
| Sjögren’s syndrome | Case-control | 1 | 1.38 (1.15-1.66) | 0.001* | NA | NA |
|  | Cohort | 4 | 1.33 (1.20-1.48) | <0.001* | 65.0 | 0.022 |
| Rheumatoid arthritis | Case-control | 2 | 0.60 (0.41-0.88) | 0.009* | 46.0 | 0.174 |
|  | Cohort | 3 | 0.92 (0.63-1.35) | 0.685 | 96.2 | <0.001 |
| Gout | Case-control | 4 | 0.92 (0.79-1.06) | 0.243 | 76.3 | 0.005 |
|  | Cohort | 5 | 1.03 (0.94-1.14) | 0.520 | 85.9 | <0.001 |

CI, confidence interval; RR, relative risk; * Statistically significant differences

**Table S4** The results of the subgroup analysis based on effect size.

| **Diseases** | **Subgroups** | **Number of studies** | **RR (95% CI)** | **P-value** | **I^2^ (%)** | **P for heterogeneity** |
| --- | --- | --- | --- | --- | --- | --- |
| Ankylosing spondylitis | OR | 2 | 1.30 (1.05-1.61) | 0.016* | 0.00 | 0.339 |
|  | HR | 2 | 1.78 (1.49-2.13) | <0.001* | 0.00 | 0.832 |
|  | SIR | 1 | 1.51 (0.86-2.65) | 0.152 | NA | NA |
| Sjögren’s syndrome | OR | 1 | 1.38 (1.15-1.66) | 0.010* | NA | NA |
|  | HR | 3 | 1.32 (1.15-1.52) | <0.001* | 79.1 | 0.008 |
|  | SIR | 1 | 2.01 (0.73-5.50) | 0.174 | NA | NA |
|  | IRR | 1 | 1.37 (1.19-1.57) | <0.001* | NA | NA |
| Rheumatoid arthritis | OR | 2 | 0.60 (0.41-0.88) | 0.009* | 46.0 | 0.174 |
|  | HR | 2 | 0.86 (0.50-1.49) | 0.594 | 97.9 | <0.001 |
|  | SIR | 1 | 1.07 (0.90-1.27) | 0.446 | NA | NA |
| Gout | OR | 4 | 0.92 (0.79-1.06) | 0.243 | 76.3 | 0.005 |
|  | HR | 3 | 1.06 (0.96-1.16) | 0.230 | 61.5 | 0.075 |
|  | RR | 3 | 1.02 (0.76-1.38) | 0.887 | 94.0 | <0.001 |
|  | IRR | 1 | 0.98 (0.89-1.07) | 0.067 | NA | NA |

CI, confidence interval; RR, relative risk; * Statistically significant differences

**Table S5** The results of the subgroup analysis based on region.

| **Diseases** | **Subgroups** | **Number of studies** | **RR (95% CI)** | **P-value** | **I^2^ (%)** | **P for heterogeneity** |
| --- | --- | --- | --- | --- | --- | --- |
| Ankylosing spondylitis | Europe | 1 | 1.51 (0.86-2.65) | 0.152 | NA | NA |
|  | Asia | 4 | 1.55 (1.28-1.89) | 0.001* | 48.9 | 0.118 |
| Sjögren’s syndrome | Europe | 1 | 2.01 (0.73-5.50) | 0.174 | NA | NA |
|  | Asia | 4 | 1.33 (1.22-1.46) | <0.001* | 64.6 | 0.023 |
| Rheumatoid arthritis | Europe | 3 | 0.74 (0.47-1.15) | 0.183 | 85.0 | 0.001 |
|  | Asia | 2 | 0.86 (0.50-1.49) | 0.594 | 97.9 | <0.001 |
| Gout | Europe | 5 | 0.99 (0.86-1.13) | 0.882 | 90.2 | <0.001 |
|  | Asia | 3 | 1.05 (0.95-1.15) | 0.348 | 68.5 | 0.013 |
|  | North America | 1 | 0.70 (0.59-0.83) | <0.001* | NA | NA |

CI, confidence interval; RR, relative risk; * Statistically significant differences

**Figure S1** Forest plot of gender-subgroup analysis for association between ankylosing spondylitis and PD risk. CI, confidence interval; RR, relative risk.


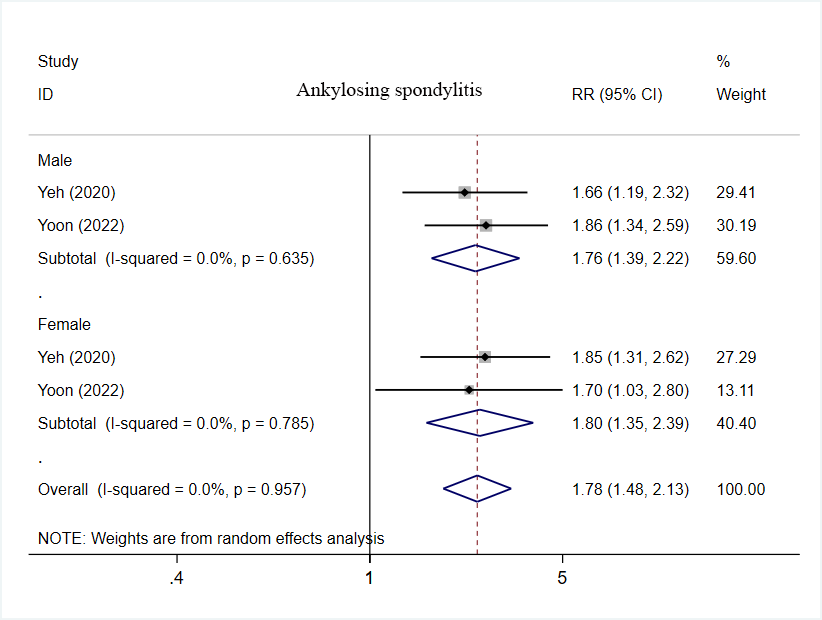


**Figure S2** Forest plot of gender-subgroup analysis for association between Sjögren’s syndrome and PD risk. CI, confidence interval; RR, relative risk.

**
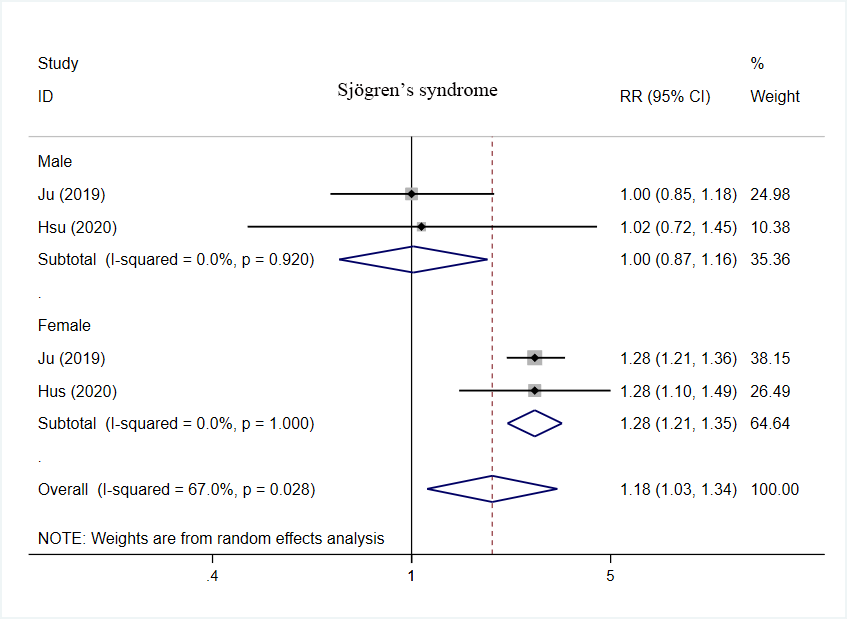
**

**Figure S3** Forest plot of gender-subgroup analysis for association between rheumatoid arthritis and PD risk. CI, confidence interval; RR, relative risk.


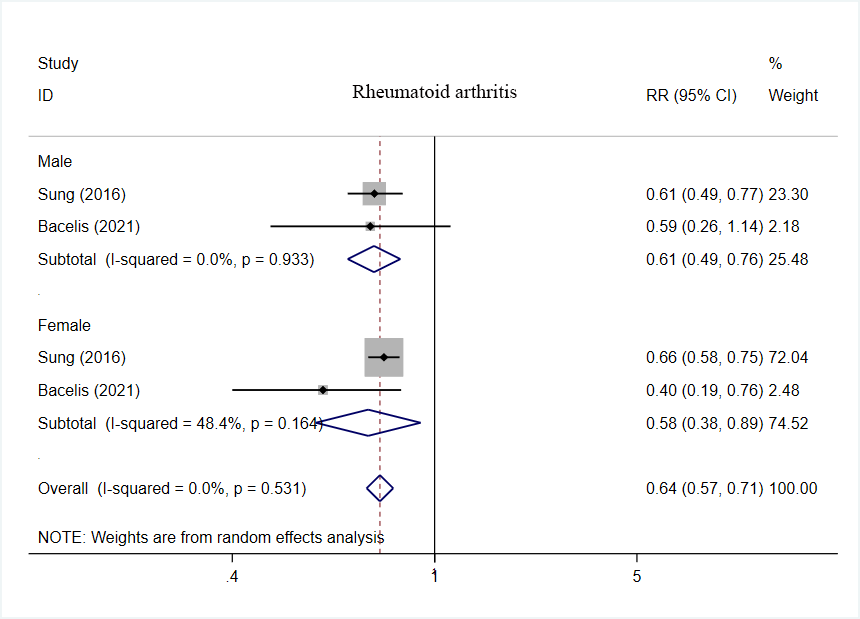


**Figure S4** Forest plot of gender-subgroup analysis for association between gout and PD risk. CI, confidence interval; RR, relative risk.
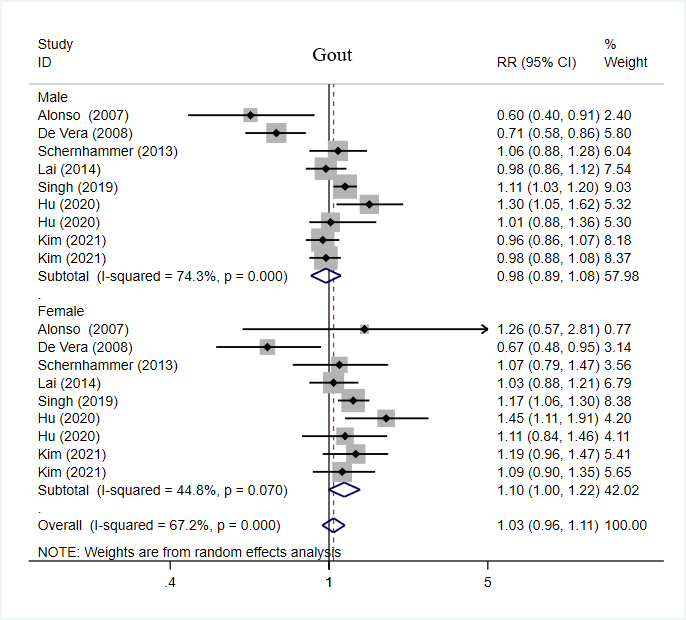


**Figure S5** Forest plot of study design’s subgroup analysis for association between ankylosing spondylitis and PD risk. CI, confidence interval; RR, relative risk.

**
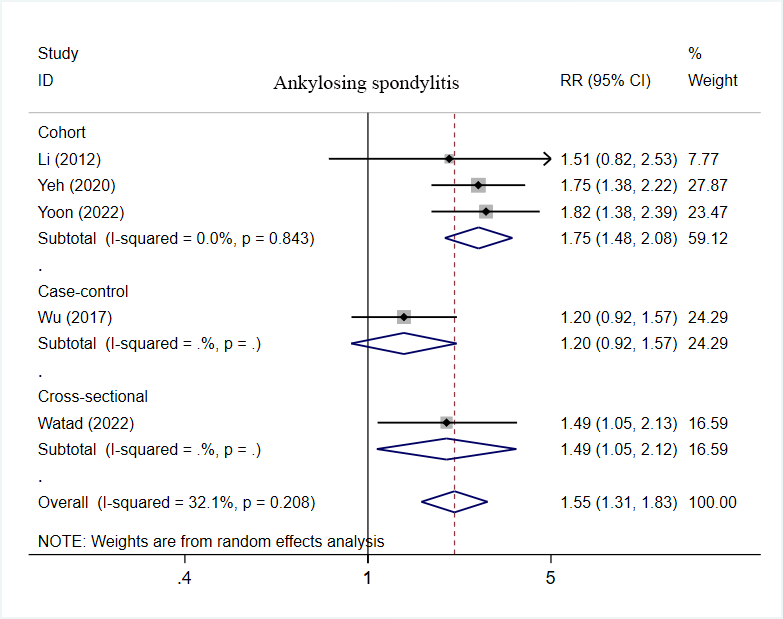
**

**Figure S6** Forest plot of study design’s subgroup analysis for association between Sjögren’s syndrome and PD risk. CI, confidence interval; RR, relative risk.


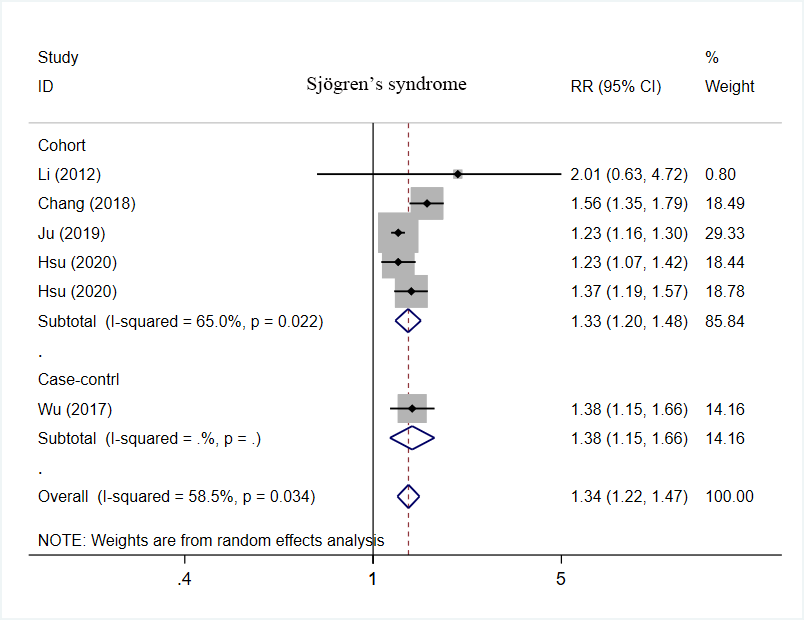


**Figure S7** Forest plot of study design’s subgroup analysis for association between rheumatoid arthritis and PD risk. CI, confidence interval; RR, relative risk.


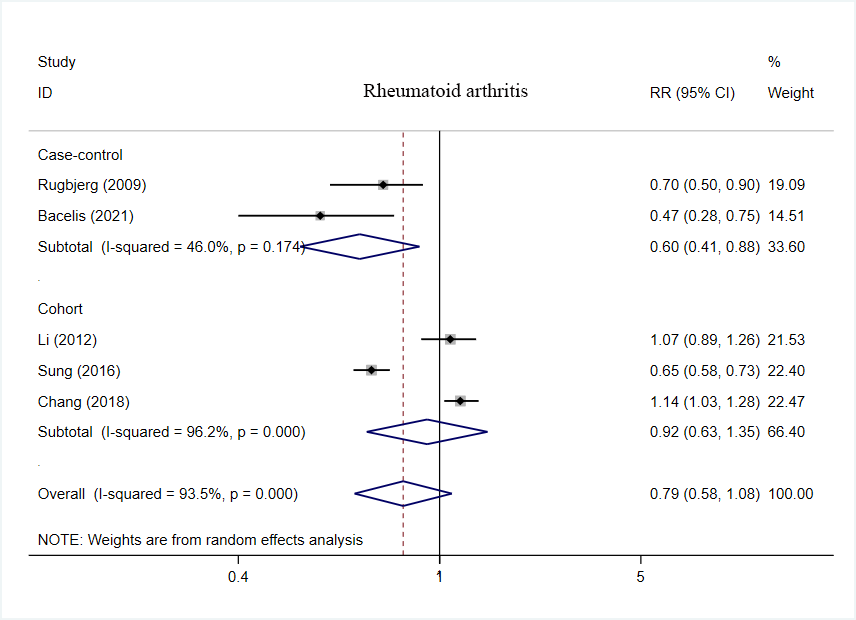


**Figure S8** Forest plot of study design’s subgroup analysis for association between gout and PD risk. CI, confidence interval; RR, relative risk.


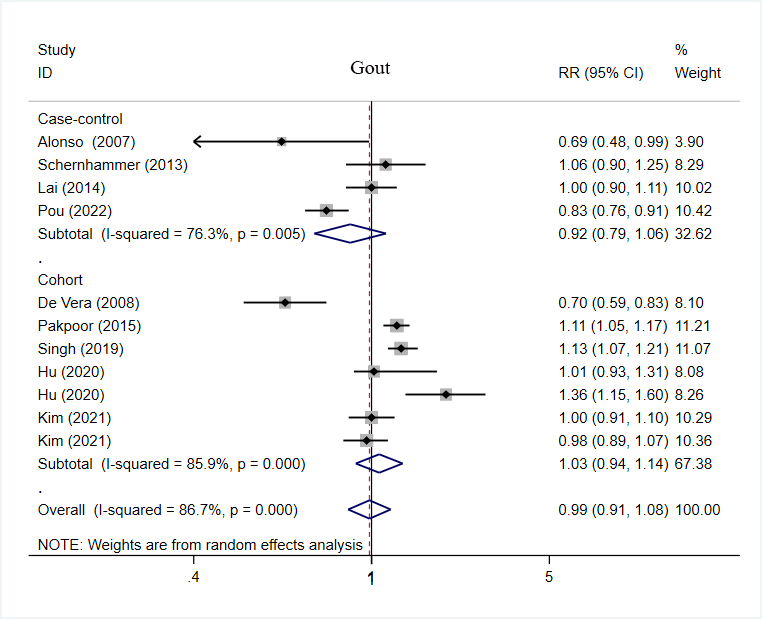


**Figure S9** Forest plot of effect size’s subgroup analysis for association between ankylosing spondylitis and PD risk. CI, confidence interval; RR, relative risk; SIR, standardized incidence ratio; OR, odds ratio; HR, hazard ratio.

**
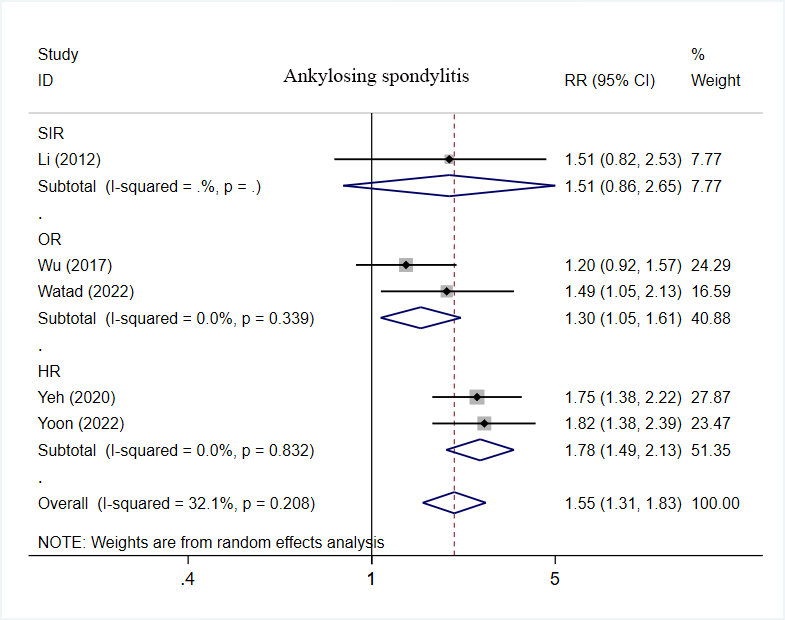
**

Figure S10 Forest plot of effect size’s subgroup analysis for association between Sjögren’s syndrome and PD risk. CI, confidence interval; RR, relative risk; SIR, standardized incidence ratio; OR, odds ratio; HR, hazard ratio; IRR, incidence rate ratio.


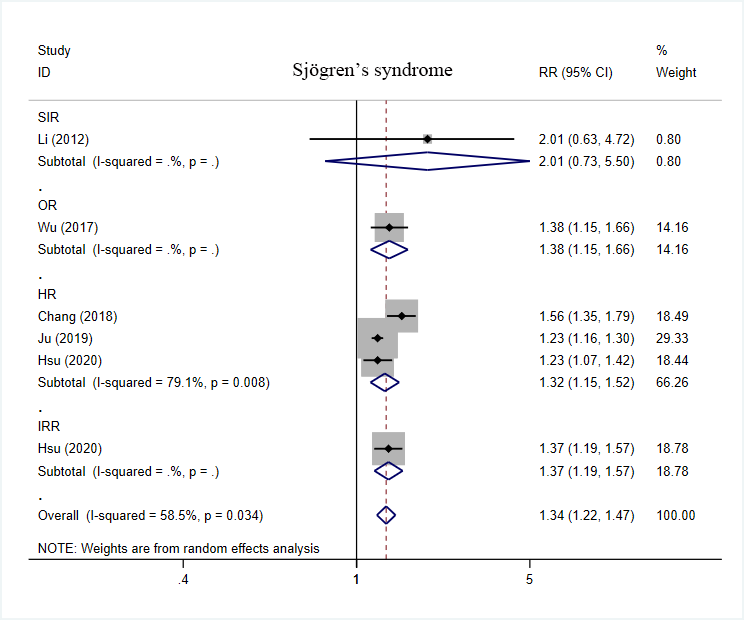


**Figure S11** Forest plot of effect size’s subgroup analysis for association between rheumatoid arthritis and PD risk. CI, confidence interval; RR, relative risk; SIR, standardized incidence ratio; OR, odds ratio; HR, hazard ratio.


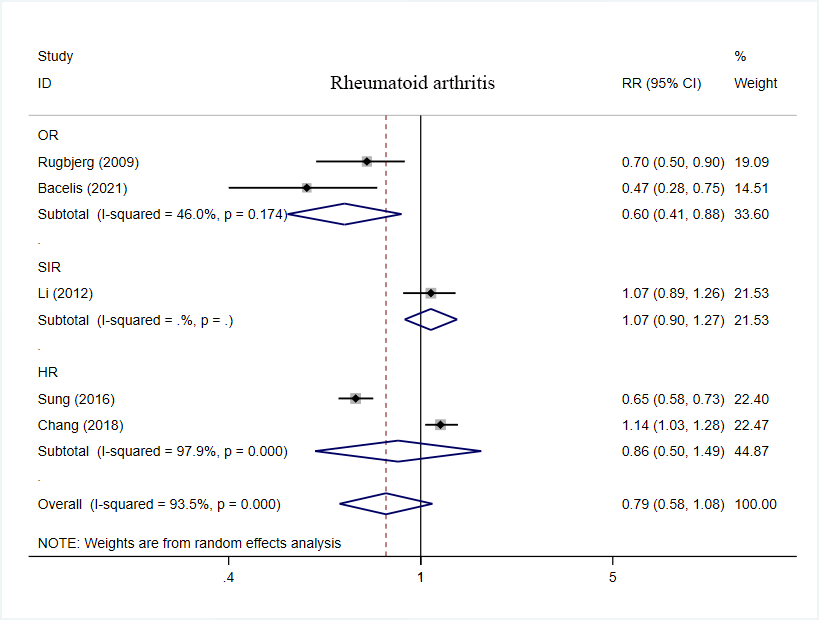


**Figure S12** Forest plot of effect size’s subgroup analysis for association between gout and PD risk. CI, confidence interval; RR, relative risk; OR, odds ratio; HR, hazard ratio; IRR, incidence rate ratio.


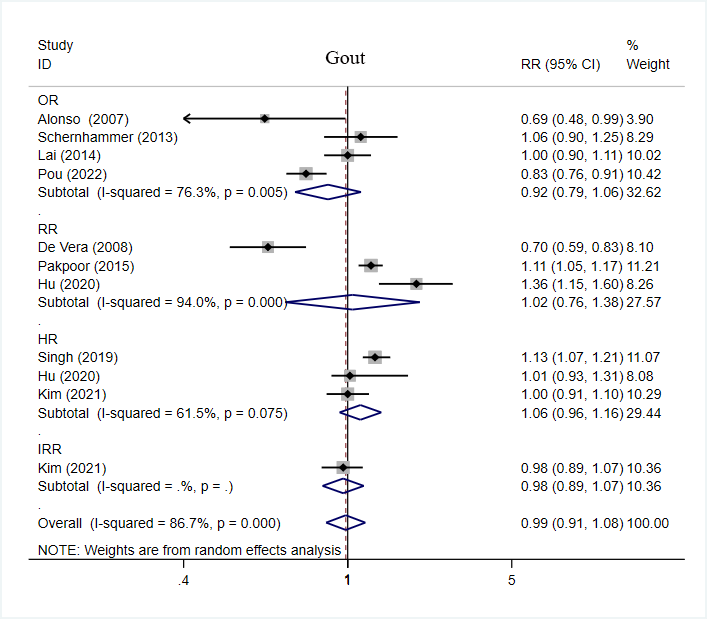


**Figure S13** Forest plot of region-subgroup analysis for association between ankylosing spondylitis and PD risk. CI, confidence interval; RR, relative risk.

**
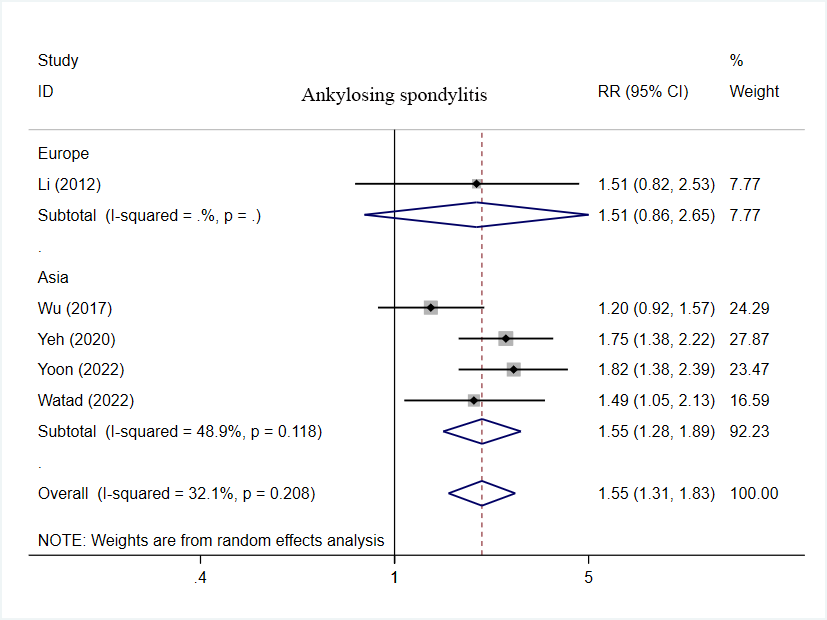
**

**Figure S14** Forest plot of region-subgroup analysis for association between Sjögren’s syndrome and PD risk. CI, confidence interval; RR, relative risk.


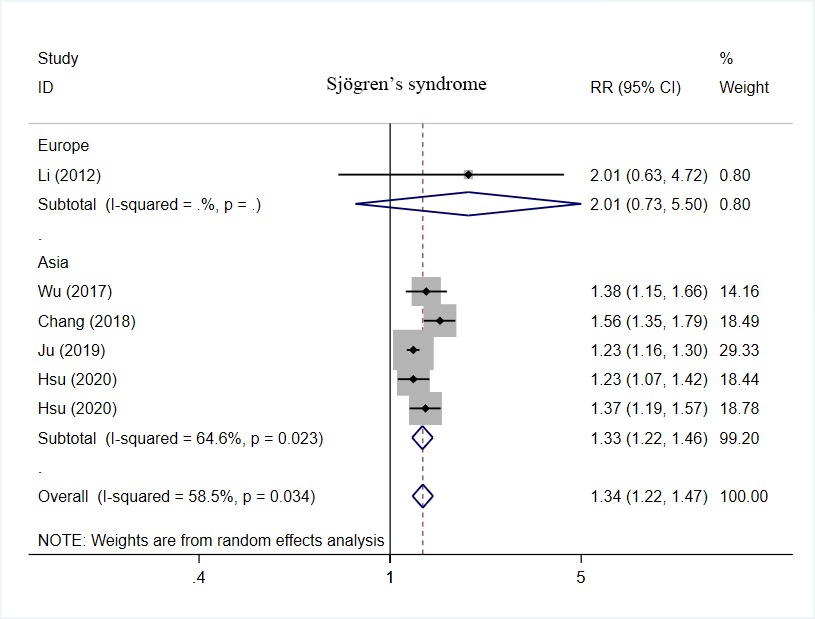


**Figure S15** Forest plot of region-subgroup analysis for association between rheumatoid arthritis and PD risk. CI, confidence interval; RR, relative risk.

**
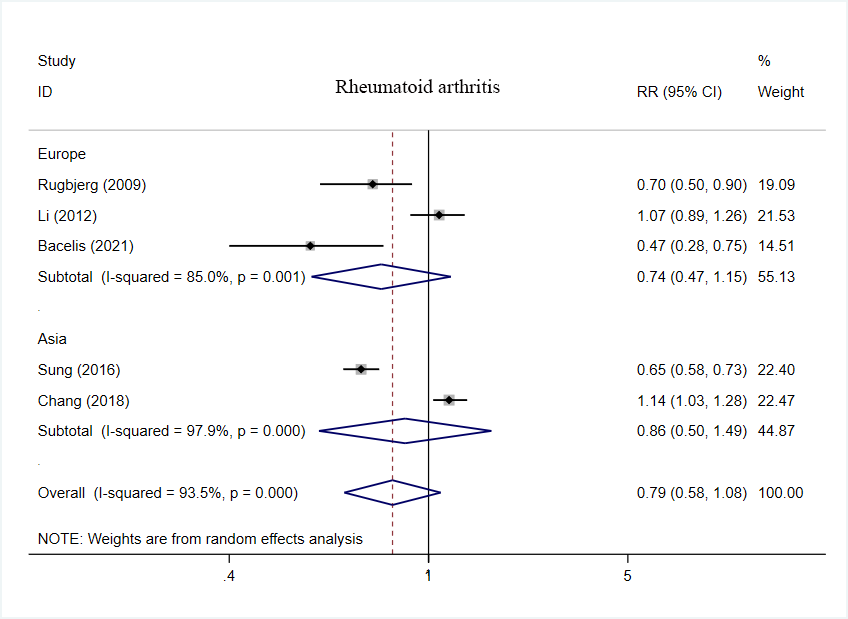
**

**Figure S16** Forest plot of region-subgroup analysis for association between gout and PD risk. CI, confidence interval; RR, relative risk.

**
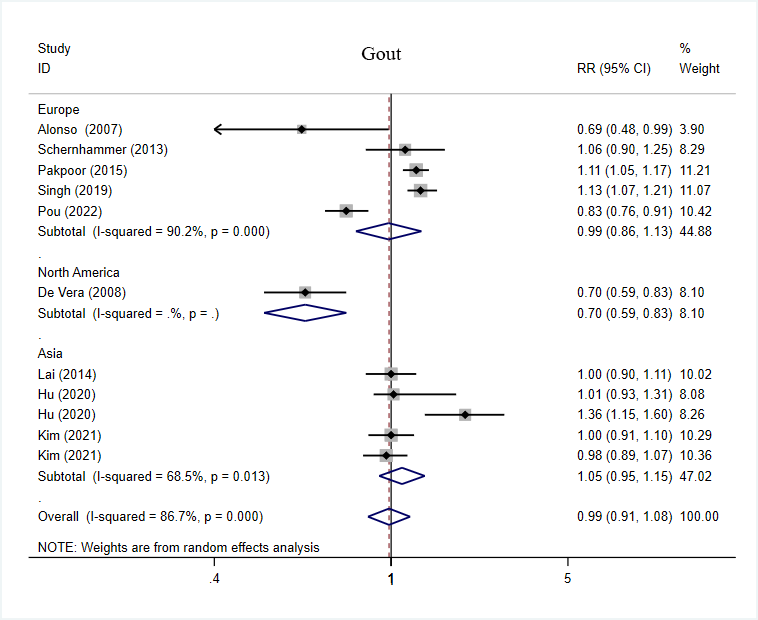
**

**Figure S17** Sensitivity analysis of studies on ankylosing spondylitis for PD risk. CI, confidence interval.


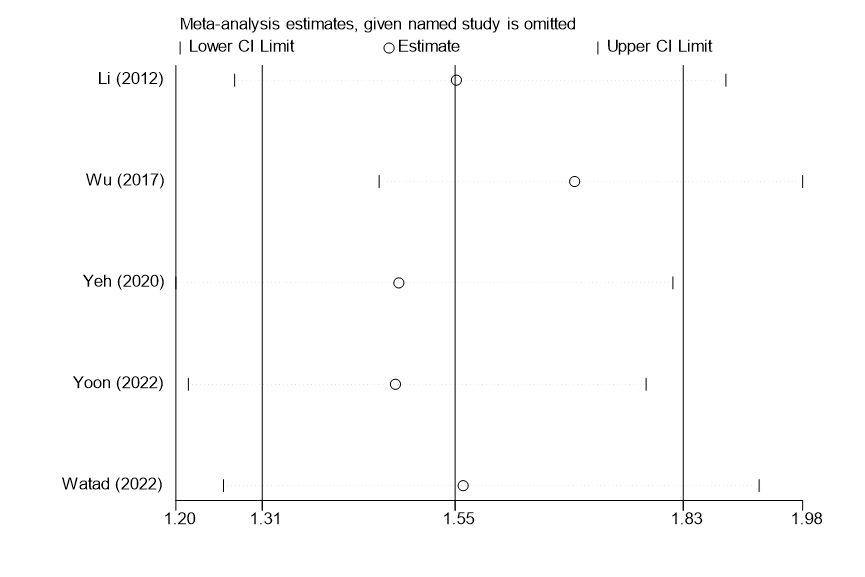


**Figure S18** Sensitivity analysis of studies on Sjögren’s syndrome for PD risk. CI, confidence interval.


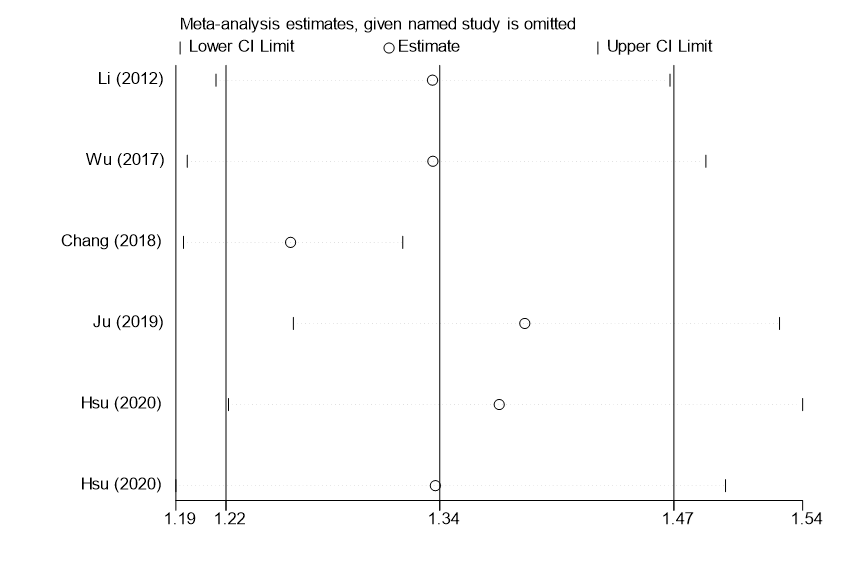


**Figure S19** Sensitivity analysis of studies on Behcet’s disease for PD risk. CI, confidence interval.

**Figure S20** Sensitivity analysis of studies on gout for PD risk. CI, confidence interval.


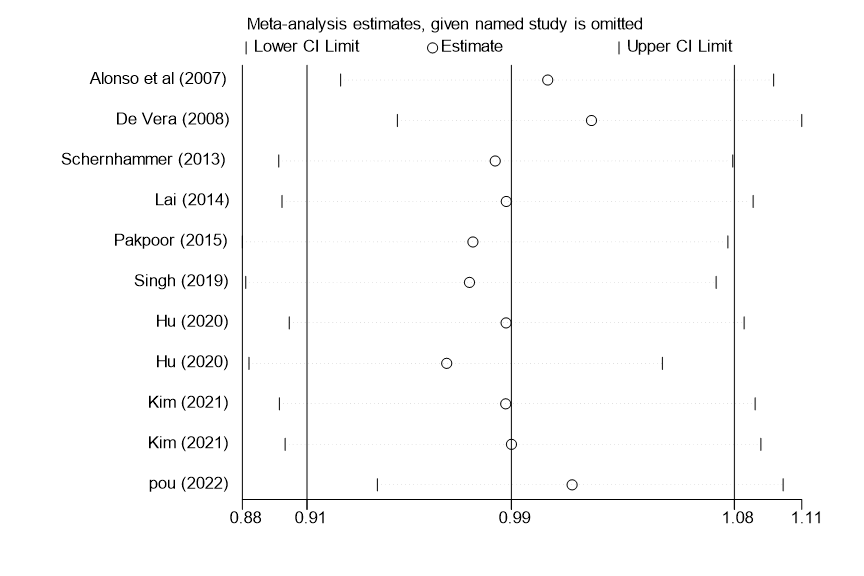


**Figure S21** Sensitivity analysis of studies on rheumatoid arthritis for PD risk.CI, confidence interval.

**Figure S22** Sensitivity analysis of studies on systemic lupus erythematosus for PD risk. CI, confidence interval.

**Figure S23** Sensitivity analysis of studies on polymyalgia rheumatica for PD risk. CI, confidence interval.

**
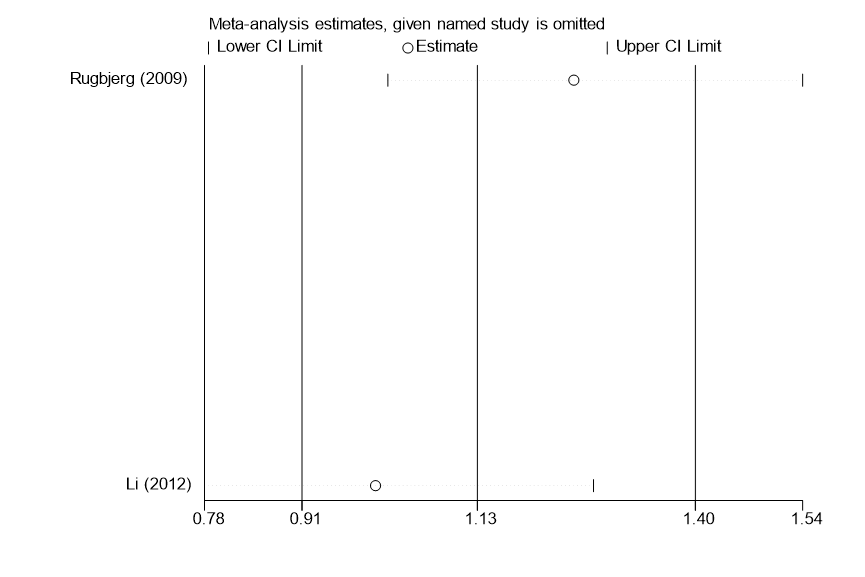
**

**Figure S24** Publication bias analysis of studies on ankylosing spondylitis for PD risk estimated by Begger’s test.

**Figure S25** Publication bias analysis of studies on Sjögren’s syndrome for PD risk estimated by Begger’s test.

**Figure S26** Publication bias analysis of studies on Behcet’s disease for PD risk estimated by Begger’s test.

**Figure S27** Publication bias analysis of studies on gout for PD risk estimated by Begger’s test.

**Figure S28** Publication bias analysis of studies on rheumatoid arthritis for PD risk estimated by Begger’s test.

**Figure S29** Publication bias analysis of studies on systemic lupus erythematosus for PD risk estimated by Begger’s test.

**Figure S30** Publication bias analysis of studies on polymyalgia rheumatica for PD risk estimated by Begger’s test.


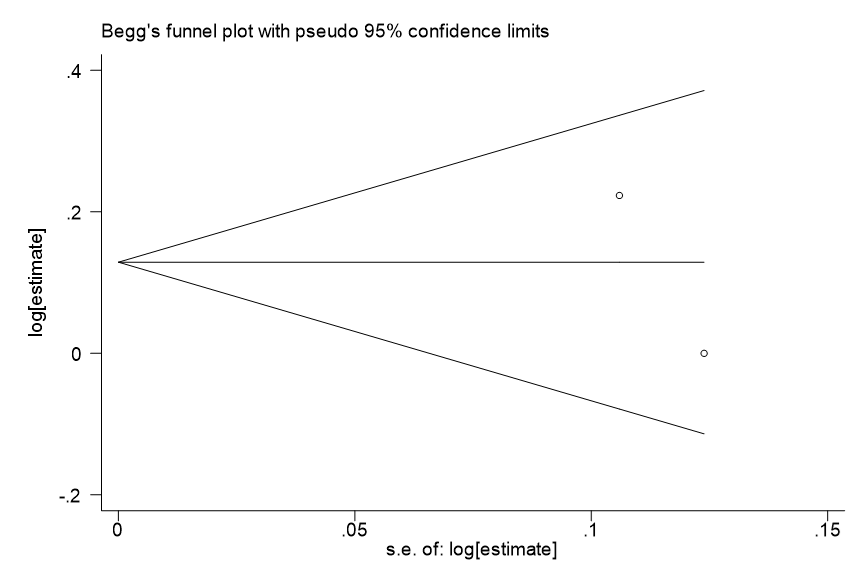

Supplement: Supplementary file 1 [file Data_Sheet_1.docx]
